# Supplementary figures and images for: Implantable photonic neural probes with 3D-printed microfluidics and applications to uncaging
Source: Front Neurosci. 2023 Jul 13;17:1213265. doi: 10.3389/fnins.2023.1213265 (PMC10373094; doi:10.3389/fnins.2023.1213265)

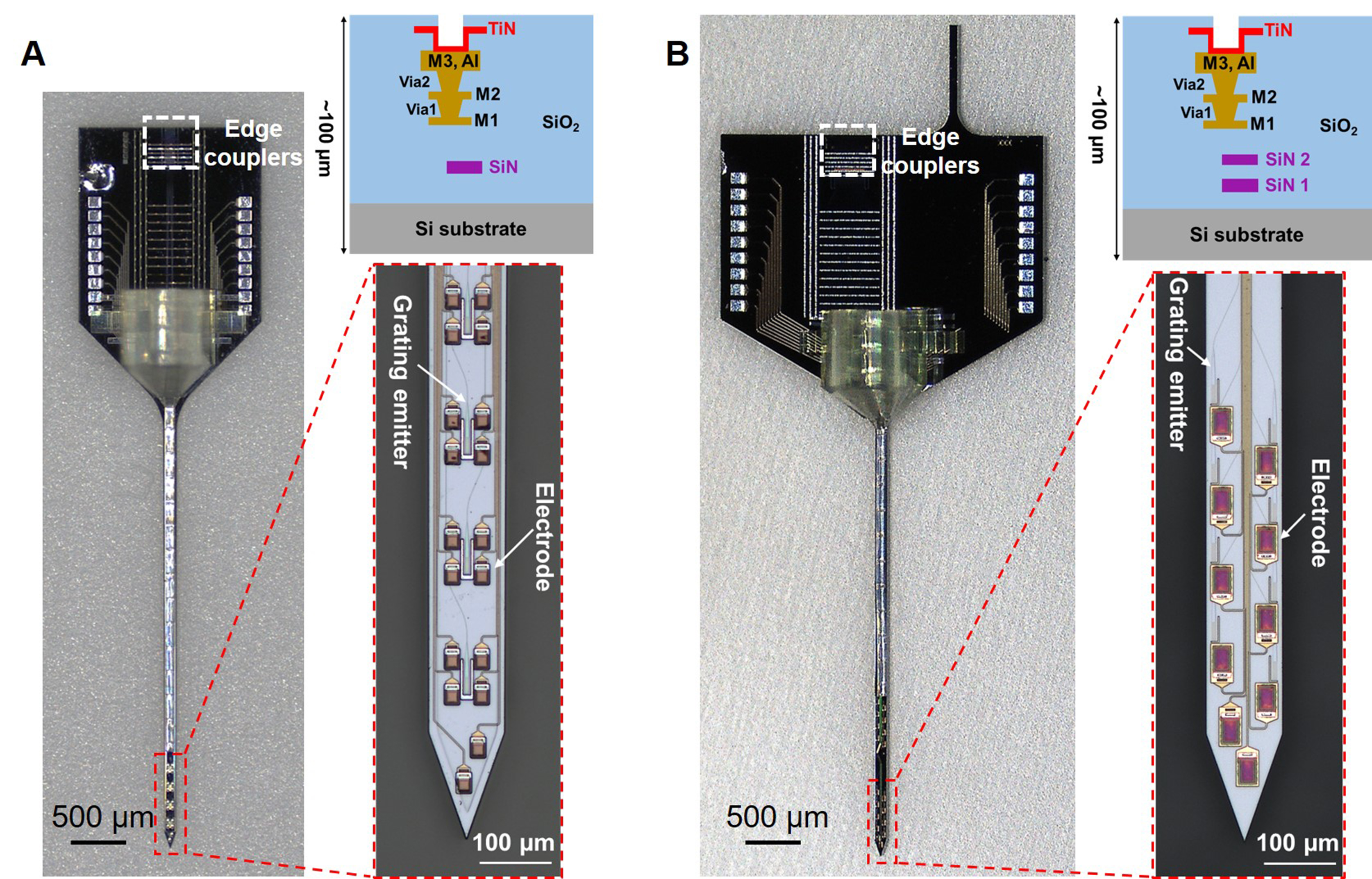

Supplement: Supplementary Figure 1 — Additional details of the neural probe designs. Comparison of (A) Probe 1 and (B) Probe 2 with optical micrographs and illustrations of the probe cross-sections. (Insets) Optical micrographs of the tips of Probe 1 and Probe 2 shanks showing grating coupler emitters and electrodes. [file Image_1.JPEG]

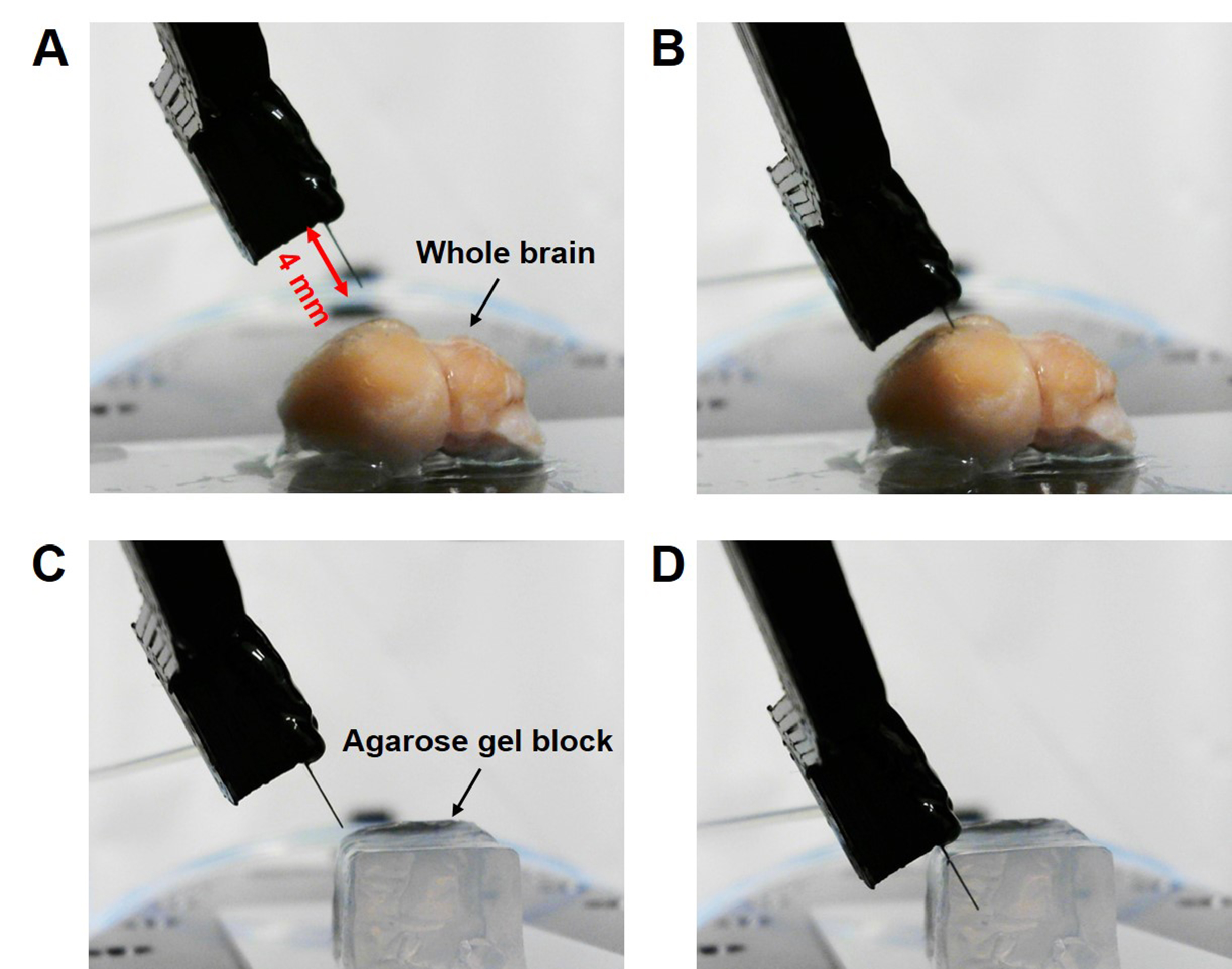

Supplement: Supplementary Figure 2 — Insertion tests of a neural probe with a 3D-printed microfluidic channel. (A) Before probe insertion into a fixed whole mouse brain. (B) Probe inserted into the fixed whole brain by about 3 mm. (C) Before probe insertion into an agarose gel block. (D) Probe inserted into the agarose gel block by about 3 mm. [file Image_2.JPEG]

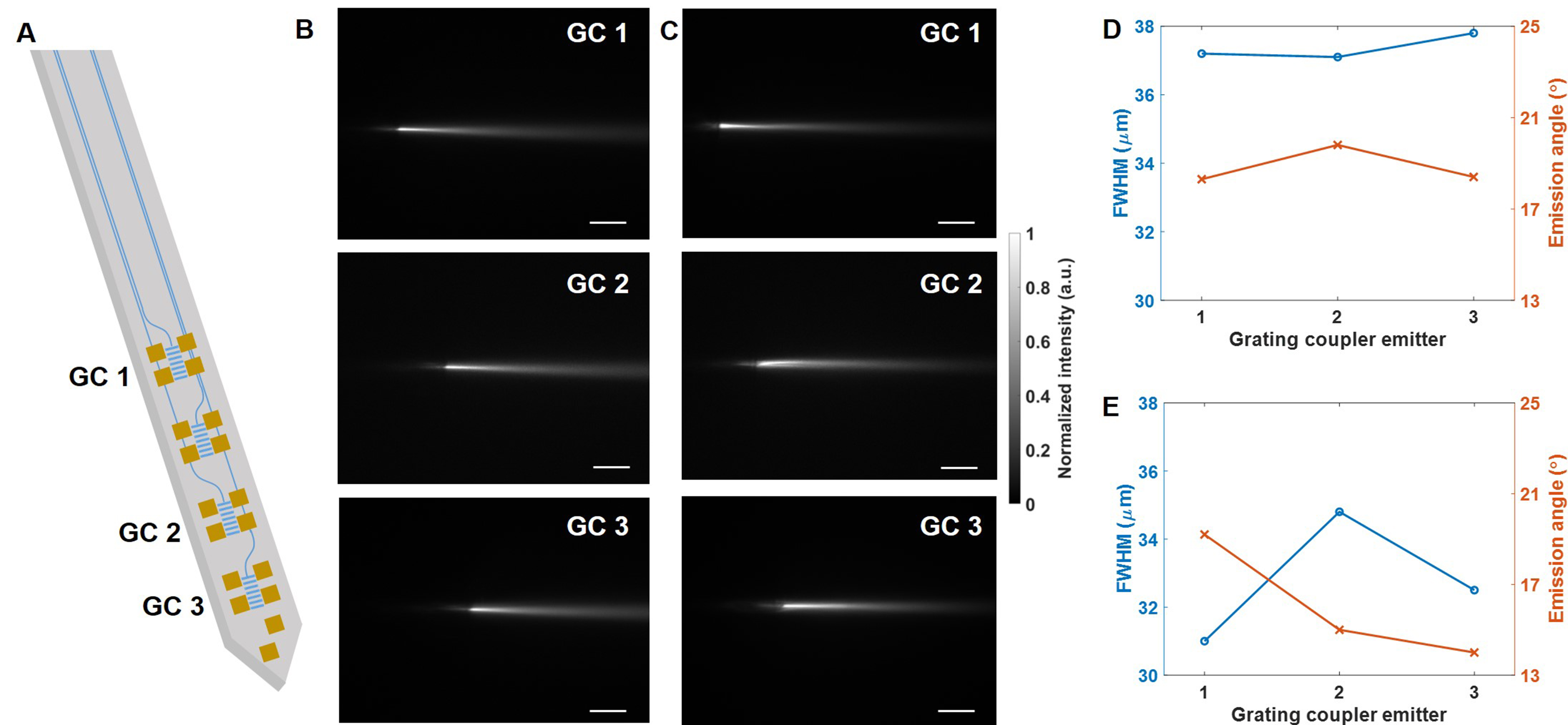

Supplement: Supplementary Figure 3 — Additional details of the measured beam profiles of Probe 1. (A) Illustration of the shank tip of Probe 1 (not to scale). Top-down fluorescence images of beam profiles from Probe 1 in (B) a fluorescein solution and (C) agarose gel. The scale bars are 100 μm. Beam profiles from grating coupler 1 (GC1), GC2, and GC3 are shown (the term “grating coupler” is synonymous with “grating emitter,” which is used elsewhere in the manuscript). Drops of fluorescein were placed on the top surface of the agarose gel and diffused into the gel, enabling fluorescence imaging of the beam profiles. Full width at half maximum (FWHM) after a 300 μm propagation distance and emission angle measurements for Probe 1 emitted beams in (D) fluorescein and (E) agarose gel. The measured FWHMs and emission angles in agarose gel have a larger variation, possibly due to impurities and structural inhomogeneity in the agarose gel block. [file Image_3.JPEG]

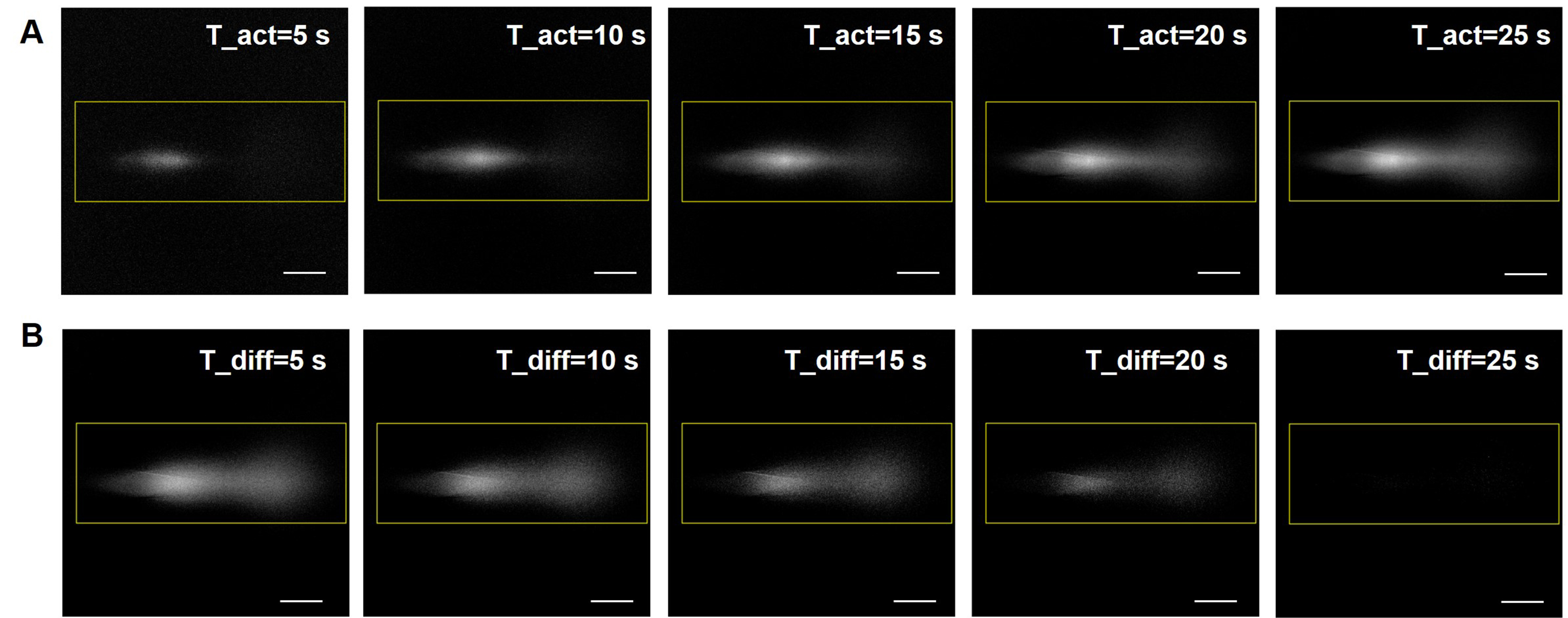

Supplement: Supplementary Figure 4 — Additional details of fluorescence intensity change (ΔF) analysis for uncaging in a CMNB-caged fluorescein solution bath (Figure 5). (A) and (B) show time-dependent fluorescence profiles with background subtraction after photoactivation and during diffusion in test 1, respectively. The scale bars are 200 μm. The region of interest (ROI) for calculation of ΔF is delineated by the yellow rectangle and was selected to avoid cropping the fluorescence profile during sequential photoactivation. ΔF was calculated as the mean pixel intensity in the ROI. Sequential micrographs of the fluorescence profiles are shown after (A) accumulated photoactivation time (T_act) and (B) diffusion time (T_diff) of 5, 10, 15, 20, and 25 s. The same ROI was applied for ΔF analysis of the three tests in Figure 5. The raw images are available at: https://doi.org/10.17617/3.5IOMQM. [file Image_4.JPEG]

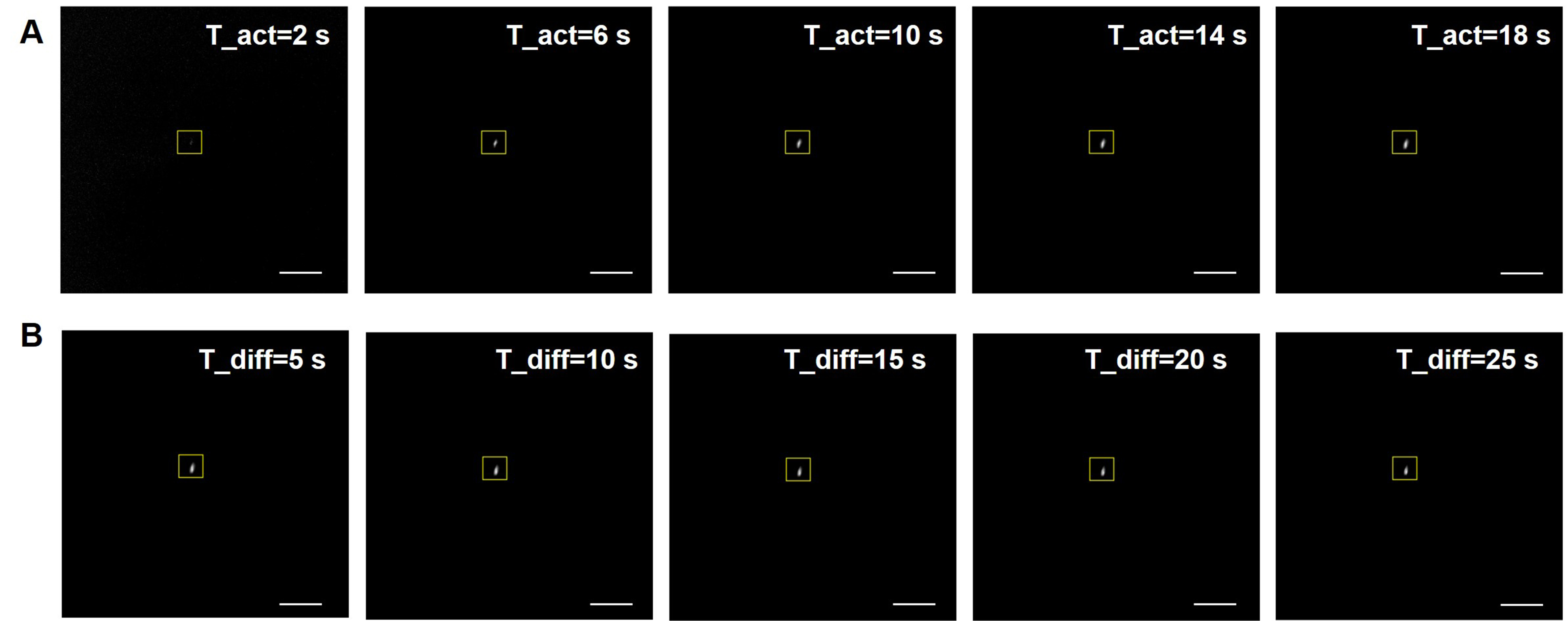

Supplement: Supplementary Figure 5 — Additional details of fluorescence intensity change (ΔF) analysis for uncaging in fixed brain tissue (Figure 7). (A) and (B) show time-dependent fluorescence profiles with background subtraction after photoactivation and during diffusion in test 1, respectively. The scale bars are 200 μm. The ROI was selected to include the fluorescence profile during sequential photoactivation. Sequential micrographs of the fluorescence profiles are shown with time stamps labeled accordingly. The same ROI was applied for ΔF analysis of the three tests in Figure 7. The raw images are available at: https://doi.org/10.17617/3.5IOMQM. [file Image_5.JPEG]

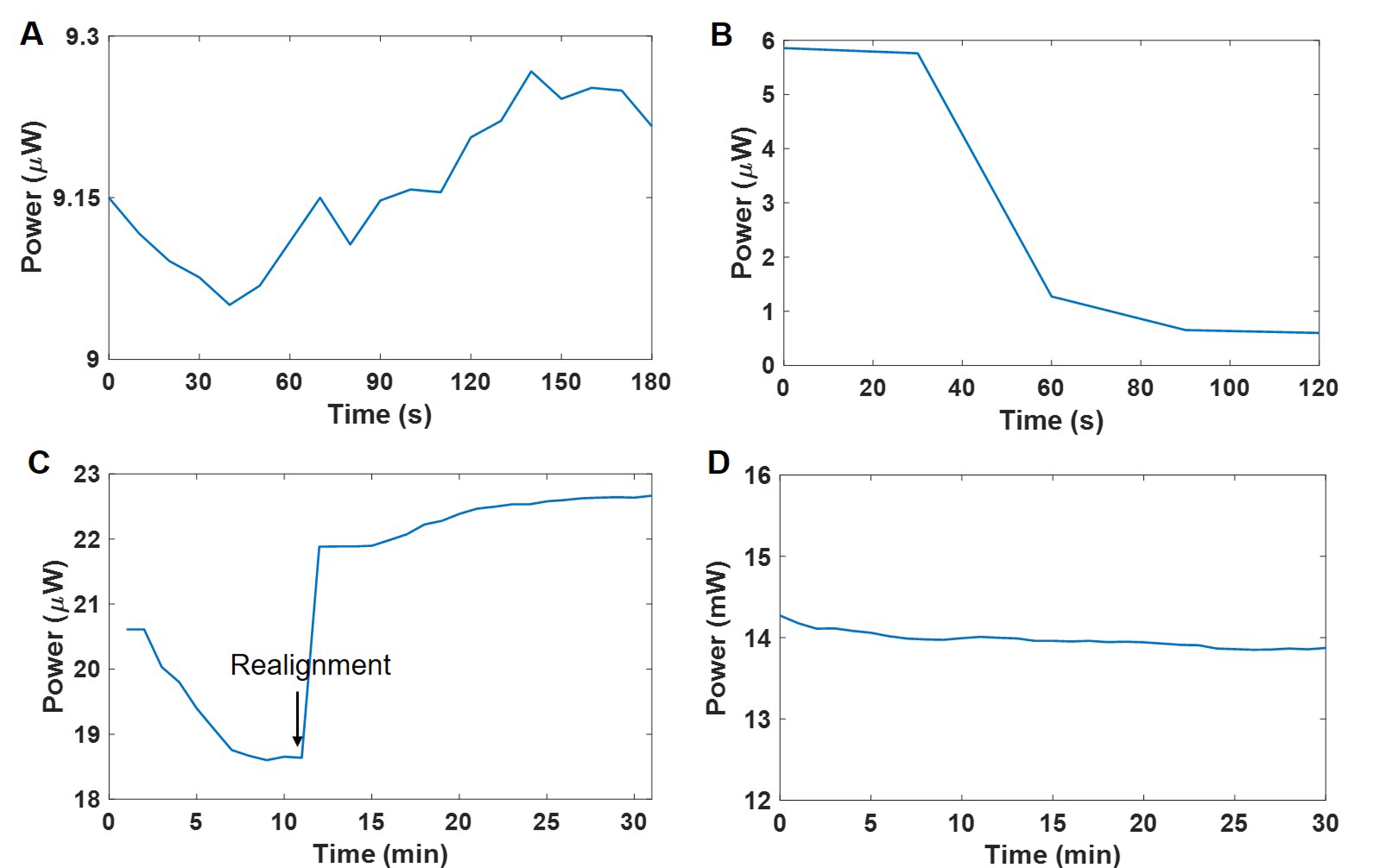

Supplement: Supplementary Figure 6 — Optical power stability test at 405 nm. Emission power of a Probe 2 grating coupler emitter corresponding to (A) −25 dB and (B) −32 dB optical transmission (with transmission defined as the ratio of emitted power from the grating coupler and input power to the optical scanning system). (C) Emission power of a grating coupler emitter from a neural probe before packaging. The neural probe under test was extracted from the same wafer as Probe 2, and 405-nm laser light was coupled to the probe chip from a single mode fiber. The alignment was re-adjusted at t=11 min. (D) Output power of a core of the multicore optical fiber, without attachment to a neural probe. [file Image_6.JPEG]
